# Is now the time to rethink risk thresholds in cancer referral guidelines?

**DOI:** 10.3399/BJGP.2025.0671

**Published:** 2025-12-01

**Authors:** Brian D Nicholson, Garth Funston, Charlotte Williamson, Samantha Leigh Harrison, Gary A Abel

**Affiliations:** 1 Nuffield Department of Primary Care Health Sciences, University of Oxford, Oxford, UK; 2 Centre for Cancer Screening Prevention and Early Diagnosis, Wolfson Institute of Population Health, Queen Mary University of London, London, UK; 3 Cancer Research UK, London, UK; 4 University of Exeter Medical School, University of Exeter, Exeter, UK

## Background

Around two thirds of patients with cancer diagnosed in England first present to primary care^1^ so encouraging timely referral of those with signs and symptoms of possible cancer is crucial to improve patient outcomes.

Cancer referral guidelines play a key role in supporting healthcare professionals to identify individuals with suspected cancer in a timely and consistent manner. Most UK guidelines use risk thresholds to determine which patients warrant urgent suspected cancer investigation or referral. These thresholds are based on the positive predictive value (PPV) of signs and symptoms alone or in combination.

Over the last decade, there has been a marked increase in cancer research developing alternative approaches to quantifying the cancer risk in patients attending primary care.

The 2015 update of the National Institute for Health and Care Excellence (NICE) NG12 guideline,^2^ used in England and Wales, introduced a 3% risk threshold to underpin their recommendations for urgent suspected cancer referral with a few exceptions (such as children’s and young people’s cancers, and investigations accessed in primary care). This was a reduction from the previous 5% threshold and was based on clinical consensus balancing diagnosing more cancers in a timely manner against overburdening the health service and potential implications for individuals (for example, unnecessary anxiety).^2^ Robust evaluation of the impact of the threshold reduction is lacking, except for some research noting minimal changes to diagnostic intervals.^3,4^

## Challenges with the current approach

Over the last decade, there has been a marked increase in cancer research developing alternative approaches to quantifying the cancer risk in patients attending primary care. This growing evidence base presents an opportunity to refresh and refine our approach to patient selection for further cancer investigation.

The approach used to quantify risk can have major implications for the size of an eligible cohort. A population-based risk estimate (such as PPV) of 3% will lead to more patients being eligible for an urgent cancer pathway compared to an individualised risk estimate of 3%, as the population-based estimate represents an average risk including some people with a very low, and very high risk of cancer. An individualised approach may allow more efficient use of health system resources, and improved outcomes.

Improved understanding of primary care tests and the development of prediction models can enable more tailored risk-based triage.

Additionally cancer pathways differ in terms of investigation type and sequencing. Applying the same risk threshold across all pathways may not be optimal from either an outcome or health economic perspective. For example, pathways that incorporate relatively cheap investigations (such as FIT, chest x-ray), and come with minimal harms to the patient could justify implementing a lower threshold for referral, and conversely a higher risk threshold used for more expensive investigations with a higher risk of harm (for example, endoscopy). Ideally, these tests will be used in sequence with a simple triage test in primary care selecting higher risk patients for a more expensive specialist test (such as FIT triage for colonoscopy).

## Tailored approaches to risk quantification

Improved understanding of primary care tests and the development of prediction models can enable more tailored risk-based triage. While PPV is helpful when determining which symptoms warrant urgent cancer referral, it may not be the optimal approach for choosing thresholds for tests or prediction models. For example, the PPV of CA125 for ovarian cancer using the current threshold (≥35 U/mL) is 9%, but the individualised risk in a woman with a CA125 value of 35 U/mL is much lower at 1%.^5^ Using the test level at which a desired risk threshold is reached, rather than the PPV of an ‘abnormal’ test result, will better identify patients who warrant investigation. Risk also varies by factors such as age. For example, a 45-year-old woman with a slightly elevated CA125 level (38 U/mL) has an estimated risk of ovarian cancer of 0.6%, while the estimated risk in a 70-year-old with the same CA125 level is five times higher (3.1%).^6^ A recent study used an ovarian cancer prediction model to identify cost-effective risk thresholds that enable higher risk women to be referred urgently and ‘low-but-not-no-risk’ women to undergo primary care ultrasound, which could improve ovarian cancer detection and patient outcomes.^7^

Similarly, studies have found that a FIT result ≥10 µg/g (the current threshold used by NICE) equates to *a*≥3% risk of colorectal cancer. However, when combined with age, sex, platelet count, and mean cell volume the threshold at which risk exceeds 3% varies.^8^ These findings demonstrate how relatively simple prediction models can stratify individuals based on risk, and encourage more efficient use of healthcare resources.

## Evidence informed thresholds

Research examining the performance of tests or new risk models is often focused on the 3% threshold, with algorithms set to align with national thresholds. These are then compared against current approaches to determine cost-effectiveness. This inflexibility does not recognise that different pathways vary in terms of costs, potential harms of investigations, capacity for investigation, impact of delayed diagnosis, and the therapeutic benefit of subsequent treatment and can be a barrier to improvement. A more logical approach would be to use cost-effectiveness analysis to select the most appropriate threshold for the specific group or cancer site once a risk model or test is developed.

A fixed threshold may be acceptable where there is no evidence to recommend an alternative solution, but more reasoned approaches should be considered if they may lead to improvements in the pathway for patients and health systems.

## Next steps

An openness towards more nuanced risk stratification in national cancer policy and guideline revisions will prepare the health system and wider community for translation of new approaches. We are aware that the implementation of more complex models is not without social and technical challenges. However, such an approach would catalyse further research and innovation, providing a clear route to impact for researchers developing and validating risk prediction models, and help align guideline development with the evolving research landscape.
